# Lacking of palladin leads to multiple cellular events changes which contribute to NTD

**DOI:** 10.1186/s13064-017-0081-6

**Published:** 2017-03-24

**Authors:** Juan Tan, Xue-Jiao Chen, Chun-Ling Shen, Hong-Xin Zhang, Ling-Yun Tang, Shun-Yuan Lu, Wen-Ting Wu, Ying Kuang, Jian Fei, Zhu-Gang Wang

**Affiliations:** 1 State Key Laboratory of Medical Genomics, Research Center for Experimental Medicine, Rui-Jin Hospital Affiliated to Shanghai Jiao Tong University School of Medicine (SJTUSM), Building 17, No. 197, Ruijin 2nd Rd, Shanghai, 200025 China; 2 Model Organism Division, E-Institutes of Shanghai Universities, SJTUSM, Shanghai, 200025 China; 3 Shanghai Research Center for Model Organisms, Shanghai, 201203 China

**Keywords:** Palladin, Neural tube defect, Cytoskeleton, Proliferation, Differentiation, Apoptosis

## Abstract

**Background:**

The actin cytoskeleton-associated protein palladin plays an important role in cell motility, morphogenesis and adhesion. In mice, Palladin deficient embryos are lethal before embryonic day (E) 15.5, and exhibit severe cranial neural tube and body wall closure defects. However, the mechanism how palladin regulates the process of cranial neural tube closure (NTC) remains unknown.

**Methods:**

In this paper, we use gene knockout mouse to elucidate the function of palladin in the regulation of NTC process.

**Results:**

We initially focuse on the expression pattern of *palladin* and found that in embryonic brain, *palladin* is predominantly expressed in the neural folds at E9.5. We further check the major cellular events in the neural epithelium that may contribute to NTC during the early embryogenesis. Palladin deficiency leads to a disturbance of cytoskeleton in the neural tube and the cultured neural progenitors. Furthermore, increased cell proliferation, decreased cell differentiation and diminished apical cell apoptosis of neural epithelium are found in palladin deficient embryos. Cell cycle of neural progenitors in *Palladin* ^*-/-*^ embryos is much shorter than that in wt ones. Cell adhesion shows a reduction in *Palladin* ^*-/-*^ neural tubes.

**Conclusions:**

*Palladin* is expressed with proper spatio-temporal pattern in the neural folds. It plays a crucial role in regulating mouse cranial NTC by modulating cytoskeleton, proliferation, differentiation, apoptosis, and adhesion of neural epithelium. Our findings facilitate further study of the function of palladin and the underlying molecular mechanism involved in NTC.

**Electronic supplementary material:**

The online version of this article (doi:10.1186/s13064-017-0081-6) contains supplementary material, which is available to authorized users.

## Background

Neurulation is the process that gives rise to the central nervous system, whereby the neural plate undergoes bending, elevating, fusion and remodeling to form the neural tube [1, 2]. In the mouse, neurulation is divided into two phases, primary and secondary neurulation. The brain and most of the spinal cord are created during primary neurulation, while the rest of the spinal cord is created during secondary neurulation. In primary neurulation, NTC is typically initiated at the midline of the hindbrain/cervical boundary, which is termed closure 1. Then neural tube fusion proceeds bi-directionally, toward both the hindbrain and the spinal cord. NTC also initiates at the midbrain/forebrain boundary and the rostral end of the forebrain, these two initiation points are designated closure 2 and closure 3, respectively. Then closure 2 proceeds caudally and rostrally, closure 3 proceeds caudally. After closure 2 meets closure 1 at the midbrain/hindbrain neuropore, and closure 3 meets closure 2 at the anterior neuropore, the whole neural tube closes completely [1, 3]. With the contribution of genetic and environmental factors, neural tube may fail to close, causing neural tube defects (NTDs) [4]. NTDs are the second most common birth defects with an incidence of 1:1000 in human births, after congenital heart defects [3]. In terms of occurring at different axial levels, there are three types of NTDs: exencephaly, spina bifida and craniorachischisis, representing the cranial region NTDs, spinal cord NTDs and NTDs along the entire body axis, respectively [5].

Animal models ranging from flies to mice have been used to explore the mechanisms underlying NTC [6–10]. By 2009, the number of mouse mutants and strains with NTDs exceeds 240, including 205 specific genes, 30 unidentified genes, and 9 multifactorial strains. These genes encode many diverse proteins that participate in cellular functions and biochemical pathways [11]. With in toto live imaging of mouse morphogenesis technology, the NTC process can be observed intuitively [12, 13]. Cellular events involved in NTC have been clarified, including actin organization, proliferation, differentiation, apoptosis, adhesion and so on [9, 10, 14]. However the molecular mechanisms behind the complex process remain poorly understood.

*Palladin* was first found to be one of genes whose expressions were up-regulated in acute promyelocytic leukemia cell line NB4 with all-trans retinoic acid (ATRA) treatment [15]. Shortly thereafter, it was reported to colocalize with α-actinin in stress fibers [16]. To study the function of palladin in vivo, we previously generated *palladin* gene knockout mice through homologous recombination, and observed NTD in palladin deficient embryos which died before E15.5 [17]. We demonstrated that peripheral nerves were not significantly stunted and neurite outgrowth was not impaired in *Palladin* ^*-/-*^ embryos [18]. However the reason that deletion of palladin causes NTD was still unknown. Over the past years, research on palladin has been focused on its interacting proteins [19, 20], its role in tumorigenesis and tumor invasion [21–24]. There are no other but our previous reports on studying its role in NTC. With the gene knockout mouse model, we have the advantages to study the role that palladin plays during NTC process. In this article, we describe that *palladin* expresses predominantly in the neural folds at E9.5 in wt mouse embryonic brain. NTD caused by palladin deficiency exhibits normal neural tube closure 1 but defective closure 2 and closure 3. Deletion of palladin leads to disrupted cytoskeleton, increased cell proliferation, decreased differentiation apoptosis, and cell adhesion in the developing neural folds. These results may partially explain the causes of NTD in *Palladin* ^*-/-*^ embryos.

## Methods

### Mice

Mice containing a heterozygous deletion of palladin were generated previously [17], and maintained on an inbred 129/Sv background under specific pathogen-free (SPF) conditions.

### Genotyping

Embryos at E9.5 were collected at somite stage 17–21, embryos at E10.5 were collected at somite stage 32–36. All experiments were performed using somite matched wt and *Palladin* ^*-/-*^ embryos. Genotype analysis of embryos and adult mice was performed as previously described [17].

### Immunofluorescence staining

Embryos were dissected and fixed in 4% paraformaldehyde overnight at 4 °C, dehydrated by sucrose series, then frozen in a Tissue Tek O.C.T. compound (Sakura Finetek, Torrance, CA), and processed to generate 10 μm frozen sections. For immunofluorescence staining, sections were treated with frozen absolute acetone for 10 min, air dried, washed with PBS and blocked with 10% normal goat serum (Jackson ImmunoResearch Laboratories) in 5% BSA/PBS for 15 min at room temperature. After incubation overnight at 4 °C with primary antibodies listed in Additional file 4: Table S1, fluorescently labeled secondary antibodies (Invitrogen) were then used and incubated for 1 h at room temperature. After antibody staining, sections were counterstained with DAPI and mounted with fluorescent mounting medium (Dako). Imaging was performed on a Nikon Eclipse 80i.

### Neural progenitors preparation

E10.5 embryonic telencephalons were microscopically dissected to separate the neuroepithelium from the mesenchyme and non-neural ectoderm. The tissue was triturated and trypsinized into single-cell suspension. Single cells were then cultured in DMEM/F-12 medium, a neural progenitor culture medium, containing 20 ng/ml EGF (R&D systems) and 20 ng/ml bFGF (R&D systems) to maintain the pluripotency. Neural progenitor cells were identified by immunofluorescence with an antibody against Nestin.

### Whole mount in situ hybridization (WISH)

WISH probes corresponding to the open reading frame were performed as previously described [25]. WISH was performed according to a standard procedure with optimization [25]. Briefly, embryos were dissected and fixed in 4% paraformaldehyde overnight at 4 °C, dehydrated and rehydrated through a graded methanol series. Embryos were then permeabilized with 10 μg/ml proteinase K in PBS for 30 min at room temperature, hybridized with 1 μg/ml digoxigenin (DIG)-UTP-labeled antisense or sense riboprobes at 68 °C. After hybridization, embryos were washed and incubated with blocking reagent for 1 h at room temperature, and then incubated with Anti-DIG-AP Conjugate (Roche) overnight at 4 °C. Signals were detected using a DIG Nucleic Acid Detection Kit (Roche). Images were taken using a Nikon SMZ 800. Primer pairs for *Palladin* isoform 2 are: sense primer: CACACACTCGGCGCACACGC; antisense primer: CAACTGGGCACCAAATACGC.

### TUNEL assay

Apoptotic cells on sections were detected using an In Situ Cell Death Detection Kit (Roche) according to the manufacturer’s protocol. Images were taken with a Nikon fluorescent microscope.

### Quantitative RT-PCR (qRT-PCR)

Total RNA was extracted from E9.5 and E10.5 embryonic brains using TriPure Isolation Reagent (Roche) according to the manufacturer’s instructions. The RNA was immediately reverse transcribed into cDNA using PrimeScript RT reagent kit (Takara) according to the manufacturer’s protocol. The process was performed under the following conditions: 37 °C for 15 min, followed by 85 °C for 5 s. SYBR Premix Ex Taq (Takara) was used for qRT-PCR with gene specific primers that amplified across exon-exon junctions. qRT-PCR was performed on an ABI Fast 7500 using the cycling conditions: 95 °C for 30 s, followed by 40 cycles of 95 °C for 5 s, 60 °C for 30 s. The transcript numbers were determined from the linear regression of the standard curves. Each qRT-PCR was performed in triplicate with normalization to *Gapdh* expression level.

### Western blot analysis

Embryonic brains were dissected and treated with radio immunoprecipitation assay (RIPA) lysis buffer (1% Nonidet P-40, 0.1% SDS and 0.5% sodium deoxycholate in PBS) with protease inhibitors mixture (Sigma). The protein concentration was assayed by BCA protein assay kit (Thermo). 50 μg of proteins were separated by SDS/PAGE. The following antibodies and dilutions were used: anti-Cyclin E polyclonal antibody (1:500, Santa cruz), anti-α-actinin monoclonal antibody (1:800, Sigma-aldrich), and anti-GAPDH monoclonal antibody (1:500, Santa cruz). The membranes were incubated with secondary antibodies (LI-COR), and then scanned with the LI-COR Odyssey imaging system.

### Cumulative BrdU Assay

Cumulative BrdU labeling in E10.5 embryos was performed as previously described [26, 27]. Briefly, pregnant female mice were intraperitoneally injected with BrdU (1 mg BrdU per 20 g body weight). A series of repeated BrdU injection was performed at an interval of two hours with the following time points: 30 min, 2 h 30 min, 4 h 30 min, 6 h 30 min, and 8 h 30 min. Embryos (*n* = 5 at each time point) were fixed 30 min after the last injection, and processed to generate frozen sections. Samples were then treated with 2 N HCl at 37 °C for 30 min, washed with PBS, incubated with 20 μg/ml proteinase K for 10 min at room temperature, and treated with 0.1% Triton X-100 for 10 min at room temperature for cell permeabilization. Treated sections were incubated with an anti-BrdU antibody at 4 °C overnight. Signals were detected using an Alexa Fluor 488 conjugated antibody. The proportion of BrdU positive nuclei of neural progenitors was used to calculate Tc (total cell-cycle length) and Ts (S phase) as previously described [26].

### Statistical analysis

The Student’s *t* test was used for statistical analyses. Standard deviation was used to measure deviation from the mean, for all experiments. For all of the statistical tests, *p* values less than 0.05 were considered statistically significant.

## Results

### Palladin deficient embryos exhibited severe cranial NTD

**Fig. 1:**
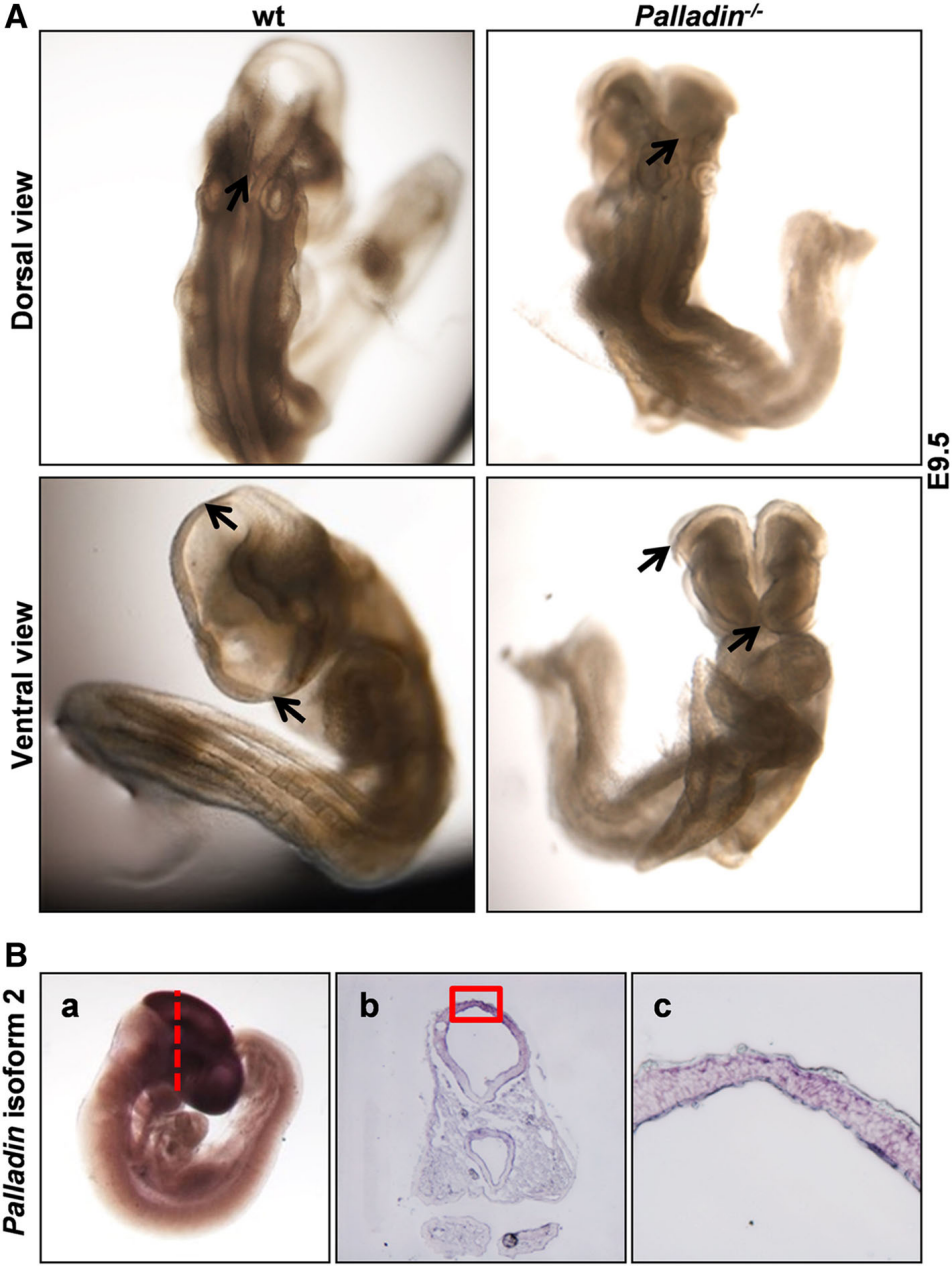
*Palladin* ^*-/-*^ embryos exhibit severe cranial neural tube defects at E9.5. **A** Whole mount embryos at E9.5 in dorsal and ventral view, anterior to the top. Neural tube closure 1 (arrows in the upper panel) forms in both wt and *Palladin* ^*-/-*^ embryos. Neural tube closure 2 (top arrows in the lower panel) and closure 3 (bottom arrows in the lower panel) fail to occur in *Palladin* ^*-/-*^ embryos. **B** Whole mount ISH of E9.5 wt embryos with *Palladin* isoform 2 probe. Note the main expression of *Palladin* in the brain region (*a*). After whole mount ISH, embryos were transected (at the indicated plane in *a*), *Palladin* locates predominantly in neural folds (*b*, *c*). The boxed region in *b* is shown at higher magnification in *c*. Abbreviations: wt-wild type, ISH-in situ hybridization

We have previously constructed *palladin* knockout mice. *Palladin* ^*-/-*^ embryos showed severe cranial NTD and died before E15.5 [17]. To further understand why palladin disruption causes cranial NTD, we collected embryos at E9.5 when *Palladin* ^*-/-*^ embryos can be distinguished from wt littermates by their failure to close the cranial neural tube. We found that cranial neural tube closure 1 initiated normally in *Palladin* ^*-/-*^ embryos, yet closure 2 and closure 3 could not occur. Therefore they displayed complete exencephaly from forebrain to hindbrain (Fig. 1A).

The embryonic central nervous system (CNS) is patterned along its left-right, dorsal-ventral, and antero-posterior axes [28]. Neural patterning is the process that provides regional identities in neural cells in accordance with their location in the neural tube. In order to investigate whether palladin deficiency impairs the neural patterning process, we used a brain mesenchyme molecular marker *Twist* and a fore/midbrain molecular marker *Otx2* to examine the development of certain parts of embryonic brain [29, 30]. The expression pattern of these two molecules was not obviously altered in *Palladin* ^*-/-*^ embryos at E9.5 (Additional file 1: Figure S1).

### *Palladin* was located predominantly in the neural folds at E9.5

We have previously determined that in E8.5 mouse embryo, *palladin* was mainly expressed in neural plate, while in E9.5 and E10.5 embryos, it showed ubiquitous expression [17]. However, the specific cell types in embryo where each isoform is expressed remain obscure. There are at least five *palladin* isoforms that produce proteins in mice due to alternative splicing and different promoter usage: isoform 1 encodes a 200 kDa protein, isoform 2 encodes a 140 kDa protein, isoform 3 and 4 encode 90–95 kDa proteins, isoform 5 encodes a 50 kDa protein [31]. Isoform 2 is the major isoform in mice brain. To identify the specific *palladin* isoforms that expressed in E9.5 mouse brains, we extracted total mRNA from E9.5 wt brains and conducted qRT-PCR using isoform-specific primers. Isoform 2 was detected to express at much higher level than the other four isoforms (Additional file 2: Figure S2). To further characterize the expression pattern of isoform 2 during cranial NTC at E9.5, WISH on E9.5 wt embryos were performed. Embryos were then processed to generate 10 μm frozen sections. We found that the expression of *Palladin* isoform 2 was restricted to the neural tube, with no expression detected in mesenchyme or non-neural ectoderm (Fig. 1B). Considering the complete exencephaly phenotype observed in *Palladin* ^*-/-*^ embryos, combined with the neural folds expression pattern of *palladin*, it would be reasonable to hypothesize that palladin might affect cranial NTC by regulating neural folds behavior. In order to confirm this hypothesis, we examined major cellular events that are essential for neural epithelium during cranial NTC. Cellular events have been inspected included cytoskeleton, proliferation, differentiation, apoptosis and adhesion.

### Cytoskeleton architecture was disturbed in palladin deficient neural folds and cultured neural progenitors

**Fig. 2:**
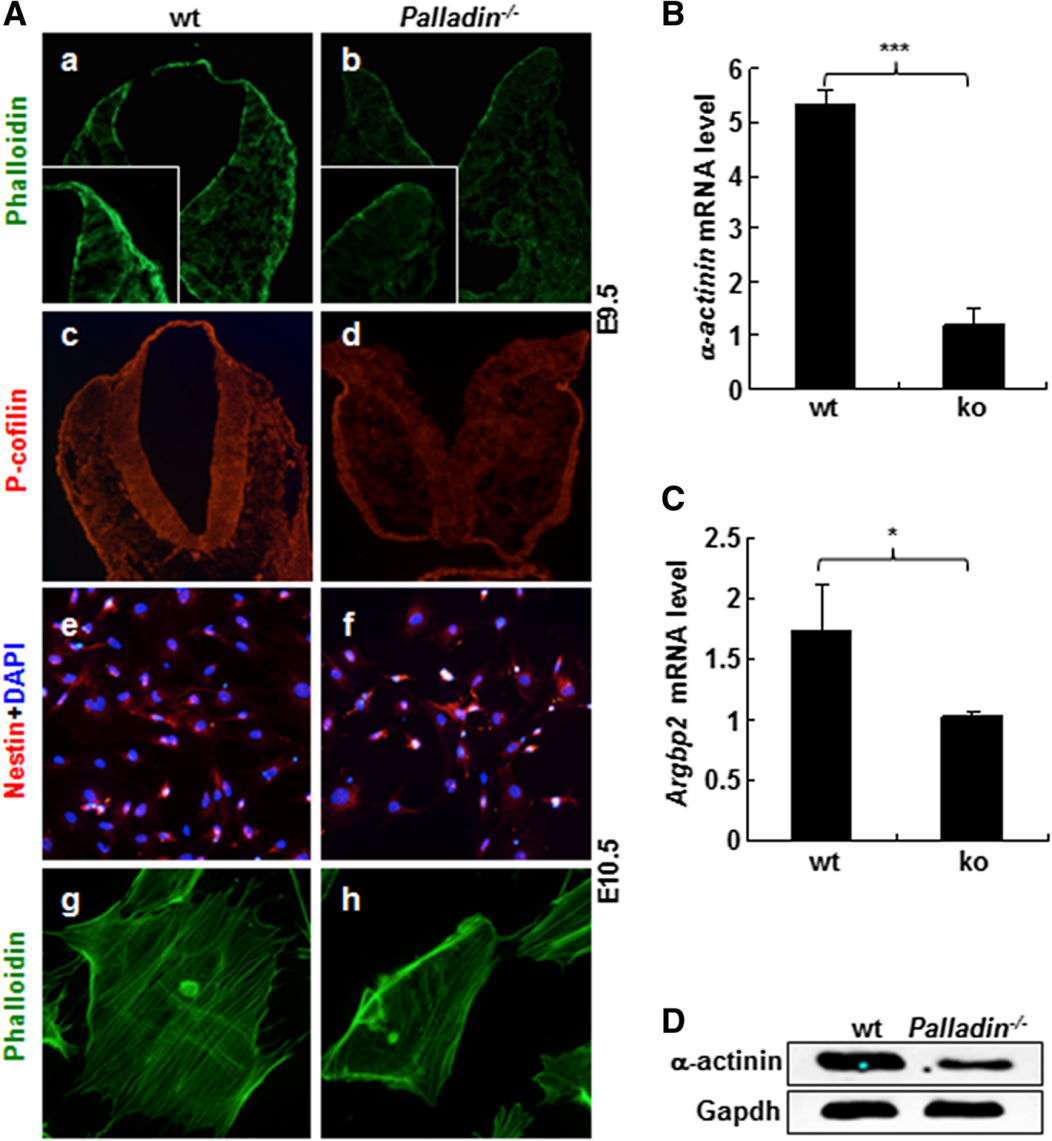
Cytoskeleton architecture is disturbed in *Palladin* ^*-/-*^ neural tube and cultured neural progenitors. (**A**) Cross-sections of embryos stained with FITC-conjugated Phalloidin and an antibody against P-cofilin, cultured neural progenitors stained with an antibody against Nestin and FITC-conjugated Phalloidin. The bundles of stress fibers in *Palladin* ^*-/-*^ neural tube (*b*, *d*) become obscure and faint compared with wt (*a*, *c*). Ratio of Nestin positive cells is over 98% in both wt and *Palladin* ^*-/-*^ neural progenitors (*e*, *f*). Stress fibers in *Palladin* ^*-/-*^ neural progenitors are atrophic (*h*) compared with wt (*g*) (**B**, **C**) mRNA levels of $\alpha$ *-actinin* (**B**) and *Argbp2* (**C**) are down-regulated in *Palladin* ^*-/-*^ embryo brains as assayed by qPCR. (**D**) $\alpha$-actinin is down-regulated in *Palladin* ^*-/-*^ embryo brains as assayed by western blotting. Magnifying power is 10X In A, *e* and *f*, while it is 20X in A, *g* and *h*. Error bars indicate SEM; *P < 0.05, ***P < 0.001

Palladin is an actin cytoskeleton-associated protein. We have previously reported that palladin deficiency resulted in weakened actin stress fibers in mouse embryonic fibroblast (MEF) cells [17]. It is possible that cytoskeleton architecture in *Palladin* ^*-/-*^ neural folds might be affected. To verify this speculation, transverse cryosections at the presumptive mid/hindbrain region from wt and *Palladin* ^*-/-*^ embryos at E9.5 were stained with an F-actin marker FITC-phalloidin to visualize actin stress fibers. The actin stress fibers (phalloidin positive) in *Palladin* ^*-/-*^ neural tube were obviously fainter than those in somite matched wt counterpart (Fig. 2A, a and b). We also stained these sections with an antibody against cytoskeleton marker P-cofilin to confirm this observation. Conformably, actin cytoskeleton architecture was more obscure in *Palladin* ^*-/-*^ neural tube compared with wt neural tube (Fig. 2A, c and d).

To determine the phenotype of cytoskeleton in *Palladin* ^*-/-*^ neural tube in vitro, neural progenitors taken from E10.5 embryonic neuroepithelium were prepared and subjected to immunostaining with Nestin (a neural stem cell and neural progenitor marker). Results showed that both wt and *Palladin* ^*-/-*^ neural progenitors were more than 98% Nestin positive (Fig. 2A, e and f), which confirmed they were truly neural progenitors. Neural progenitors were then stained with FITC-phalloidin. F-actin showed a significant decrease in E10.5 *Palladin* ^*-/-*^ neuroepithelium-derived neural progenitors, compared to that in wt neural progenitors (Fig. 2A, g and h).

In order to understand the mechanism behind the decreased cytoskeleton in *Palladin* ^*-/-*^ neural tube, total RNAs were extracted from E9.5 wt and *Palladin* ^*-/-*^ embryonic brains, and reverse transcribed into cDNAs. The mRNA expression of cytoskeleton related proteins which can interact with palladin were then detected in these two genotypic cDNAs by qRT-PCR. Genes have been detected included *α-actinin*, *Argbp2, Eps8, VASP, AKT1, Profilin, LASP1, Clp36, Spin90, Ezrin, ILKAP, LPP* and *Src* [20, 31–41]*.* The expression of both *α-actinin* and *Argbp2* showed a significant decrease in *Palladin* ^*-/-*^ embryonic brain-derived cDNAs compared to that in wt cDNAs (Fig. 2B-C). mRNA levels of *Eps8, VASP, AKT1, Profilin, LASP1, Clp36, Spin90, Ezrin, ILKAP, LPP* and *Src* showed no remarkable difference between wt and *Palladin* ^*-/-*^ brain at E9.5 (Additional file 3: Figure S3A). To confirm this finding, proteins from wt and *Palladin* ^*-/-*^ embryonic brains were prepared to perform western blot assay, α-actinin was remarkably down-regulated in *Palladin* ^*-/-*^ embryonic brains (Fig. 2D). The decreased expression of these two genes may contribute to the disturbed cytoskeleton in *Palladin* ^*-/-*^ neural tube.

### Loss of palladin resulted in increased proliferation and abnormal differentiation in neural folds

**Fig. 3:**
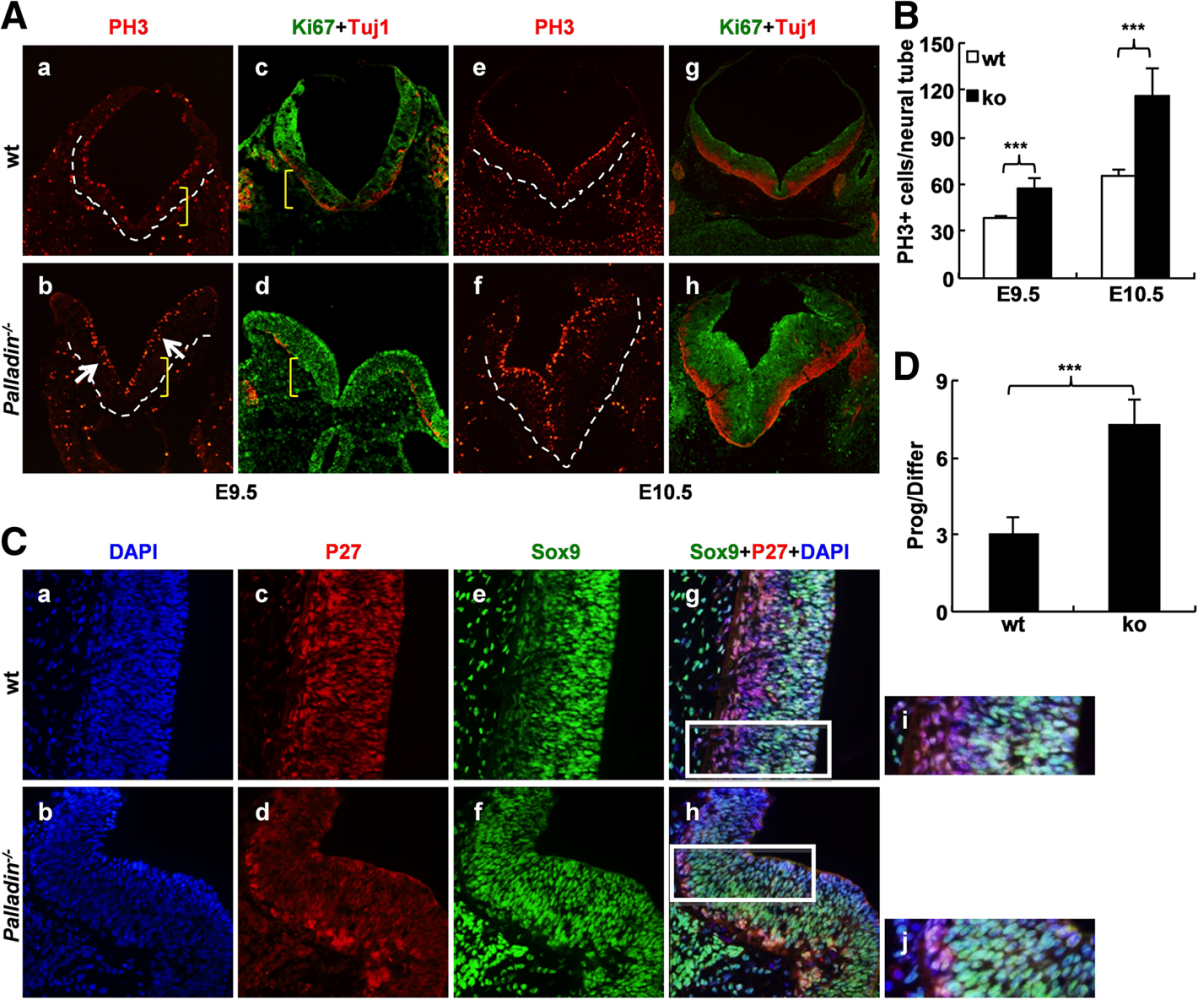
Proliferation is increased while differentiation is reduced in *Palladin* ^*-/-*^ neural tube. (**A**) Cross-sections of embryos are detected for PH3, Ki67 and Tuj1. Proliferation is increased in *Palladin* ^*-/-*^ neural tube at both E9.5 (*b*, *green* in *d*) and E10.5 (*f*, *green* in *h*). At E9.5, differentiation mainly occurs in the ventral side of wt neural tube (*red* in *c*) but dorsal side of *Palladin* ^*-/-*^ neural tube (*red* in *d*). Differentiation is decreased in the ventral side of neural tube in *Palladin* ^*-/-*^ at E10.5 (*red* in *g* and *h*). (**B**) Quantification of PH3-positive cells in the neural tube region at both E9.5 and E10.5. (**C**) E10.5 neural tubes are detected for Sox9 and P27. Neural progenitors (*green*) are increased and differentiated neurons (*red*) are reduced in *Palladin* ^*-/-*^ neural tube. The boxed region in *g* and *h* are shown at higher magnification in *i* and *j* respectively. (**D**) Quantification of the ratio of progenitors to differentiated neurons at E10.5. White dotted lines outline the neural tube, yellow brackets indicate ventral side of the neural tube, while arrows indicate abnormal proliferation in the internal area of neural tube. Error bars indicate SEM; ***P < 0.001

Cytoskeleton-associated proteins play a role in cell proliferation [42]. Previous studies noted that proper proliferation of neural progenitor cells is critical to NTC [43]. The observation that *Palladin* ^*-/-*^ embryos displayed NTD suggested that proliferation might be disrupted in *Palladin* ^*-/-*^ embryos. Therefore, transverse cryosections from wt and *Palladin* ^*-/-*^ embryos at E9.5 and E10.5 were stained with antibodies against phosphorylated-histone H3-Ser10 (PH3, a M phase marker, marking mitotic cells) and Ki67 (a late G1-M phase marker, marking all dividing cells). For PH3 staining, the number of PH3-positive cells was greatly increased in the region of neural tube at both E9.5 and E10.5 (Fig. 3A, a, b, e and f). PH3-positive cells in the neural tubes were quantitated for 3 sections of each embryo (five embryos from each genotype were stained). The average cell number was compared using unpaired two-tailed student’s *t* test between wt and *Palladin* ^*-/-*^ neural tube. It led to a significant difference in both E9.5 and E10.5 embryos (Fig. 3B). Results also showed that at E9.5, there were more PH3-positive cells in dorsal than ventral neural tube in wt embryos (Fig. 3A, a). However, in *Palladin* ^*-/-*^ embryos, the distribution of PH3-positive cells was more uniform between the dorsal and ventral aspect of the cranial neural tube (Fig. 3A, b). At E10.5, when the cranial NTC finished, proliferation was uniform along the dorso-ventral axis in both wt and *Palladin* ^*-/-*^ embryos (Fig. 3A, e and f). Moreover, at E9.5, PH3-positive cells specifically distributed at the first layer of neural epithelium at the duct edge in wt neural tube, while they could also been seen in the more internal area in *Palladin* ^*-/-*^ neural tube (arrows in Fig. 3A, a and b). The abnormal proliferation showed in *Palladin* ^*-/-*^ embryos was confirmed by Ki67 staining. Proliferation was enormously increased in *Palladin* ^*-/-*^ neural tube at both E9.5 and E10.5 (Fig. 3A, c, d, g and h).

The development of the nervous system is a very complicated process. It is not only spatially but also temporally regulated, depending on the production of functionally diverse neuronal cell types at their proper locations. Differentiation and proliferation of neural epithelium are related and interdependent. Considering the abnormally increased proliferation in *Palladin* ^*-/-*^ embryos, we speculated that differentiation might be disrupted. Therefore, we stained embryos at E9.5 and E10.5 with antibody against TuJ1 (also know as beta III Tubulin, a neuronal differentiation marker). At E9.5, in the ventral side of wt neural tube, neural progenitors began to exit the cell cycle, and gradually differentiate into neurons and glial cells, while no differentiation could be seen in *Palladin* ^*-/-*^ embryos at the same domain (Fig. 3A, c and d). Instead, differentiation was observed in the dorsal half of the neural tube in *Palladin* ^*-/-*^ embryos, which was not obvious in wt embryos (Fig. 3A, c and d). Interestingly, the area with decreased differentiation in the ventral side of neural tube was corresponding to the region of abnormally increased proliferation in *Palladin* ^*-/-*^ neural tube. Moreover, at E10.5, with more proliferation in *Palladin* ^*-/-*^ than wt embryos, differentiation declined in *Palladin* ^*-/-*^ embryos compared with wt counterparts (Fig. 3A, g and h). This unique correlated pattern of proliferation and differentiation is a confusing issue.

### Decreased cell-cycle length in palladin deficient neural progenitors

In order to further elucidate the defects of increased proliferation and decreased differentiation, cryosections from E10.5 wt and *Palladin* ^*-/-*^ embryos were stained with antibodies against Sox9 (a neural progenitor marker) and P27 (a differentiated neuron marker). The number of Sox9 positive cells and P27 positive cells in the ventral side of neural tube was quantitated respectively to represent the neural progenitors and the differentiated neurons. Results showed neural progenitors increased and differentiated neurons decreased in *Palladin* ^*-/-*^ neural tube (Fig. 3C). The ratio of proliferating neural progenitors to differentiated neurons was more than 2-fold increased in *Palladin* ^*-/-*^ embryos than that in wt controls (Fig. 3D). Also qPCR analysis of neural progenitor genes *Sox9*, *Nestin* and *Pax6* and differentiation genes *MAP2*, *Olig2* and *Neurogenin2* was performed to address the phonotype of increased proliferation and decreased differentiation, it turned out that the expression of *Sox9* increased in *Palladin* ^*-/-*^ embryos (Additional file 3: Figure S3B).

**Fig. 4:**
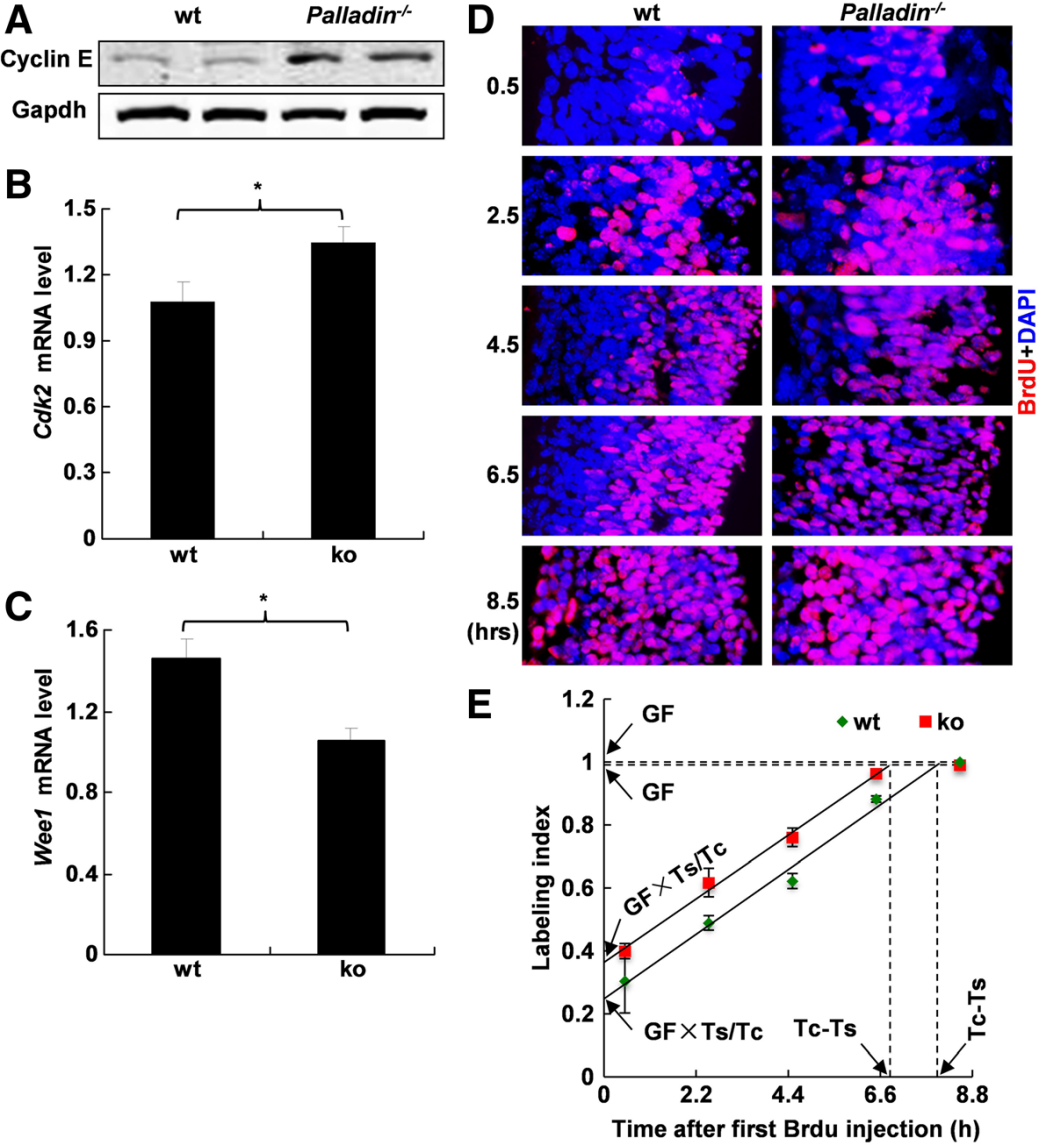
Cell cycle of *Palladin* ^*-/-*^ neural progenitors at E10.5 is shortened. **A** Expression of Cyclin E in *Palladin* ^*-/-*^ embryo brains is up-regulated at E10.5, as detected by western blotting. Expression of *Cdk2* (a cell cycle enhancer) is up-regulated (**B**) while expression of *Wee1* (a cell cycle suppressor) is down-regulated (**C**) in *Palladin* ^*-/-*^ embryo brain at E10.5, as assayed by qPCR. Error bars indicate SEM; *P < 0.05. **D** BrdU labeling of neural progenitors (*pink*) after indicated time of incubation. The proportion of BrdU-stained progenitors is higher in *Palladin* ^*-/-*^ neural progenitors at each time point (right panel) compared with wt (left panel). **E** Cell cycle of neural progenitors in wt and *Palladin* ^*-/-*^ at E10.5, Tc-Ts is 7.95 h in wt and 6.83 h in *Palladin* ^*-/-*^ neural progenitors (*n* = 5 at each time point). Error bars indicate SEM

Change in cell proliferation is often related to a change in cell cycle length. Embryonic cell cycle process is mediated by cyclins, cyclin-dependent kinases (cdks) and cyclin-related proteins [44]. In order to verify whether the increase of neural progenitors in E10.5 *Palladin* ^*-/-*^ neural tube was due to a shortened cell cycle length, we first examined the expression of cyclin E (a key positive regulator of cell cycle phase G1/S), by western blotting on embryonic brain proteins from E10.5. Cyclin E showed a significant increase in *Palladin* ^*-/-*^ embryonic brain (Fig. 4A). *Cdk2* (a cyclin E binding partner) was up-regulated and *wee1* (a negative regulator of phase G1/S) was down-regulated in E10.5 *Palladin* ^*-/-*^ brain, determined by qRT-PCR of E10.5 embryonic brain cDNA (Fig. 4B-C). Expression of *Cyclin A, Cyclin B* and *Cyclin D1* was also addressed by qRT-PCR, it showed no significant change in *Palladin* ^*-/-*^ brain (Additional file 3: Figure S3C). This data suggested a shortened G1/S length in cell cycle of neural progenitors in *Palladin* ^*-/-*^ embryos. To verify this speculation, a cumulative BrdU incorporation study was performed. At all the five time points, the proportion of BrdU positive cells was significantly higher in *Palladin* ^*-/-*^ neural tube than that in wt neural tube (Fig. 4D). In wt cranial neural tube, the total cell cycle length minus S phase length was 7.95 h, whereas in *Palladin* ^*-/-*^ siblings it was shortened to 6.83 h (Fig. 4E).

### Decreased apoptosis in neural folds after palladin disruption

**Fig. 5:**
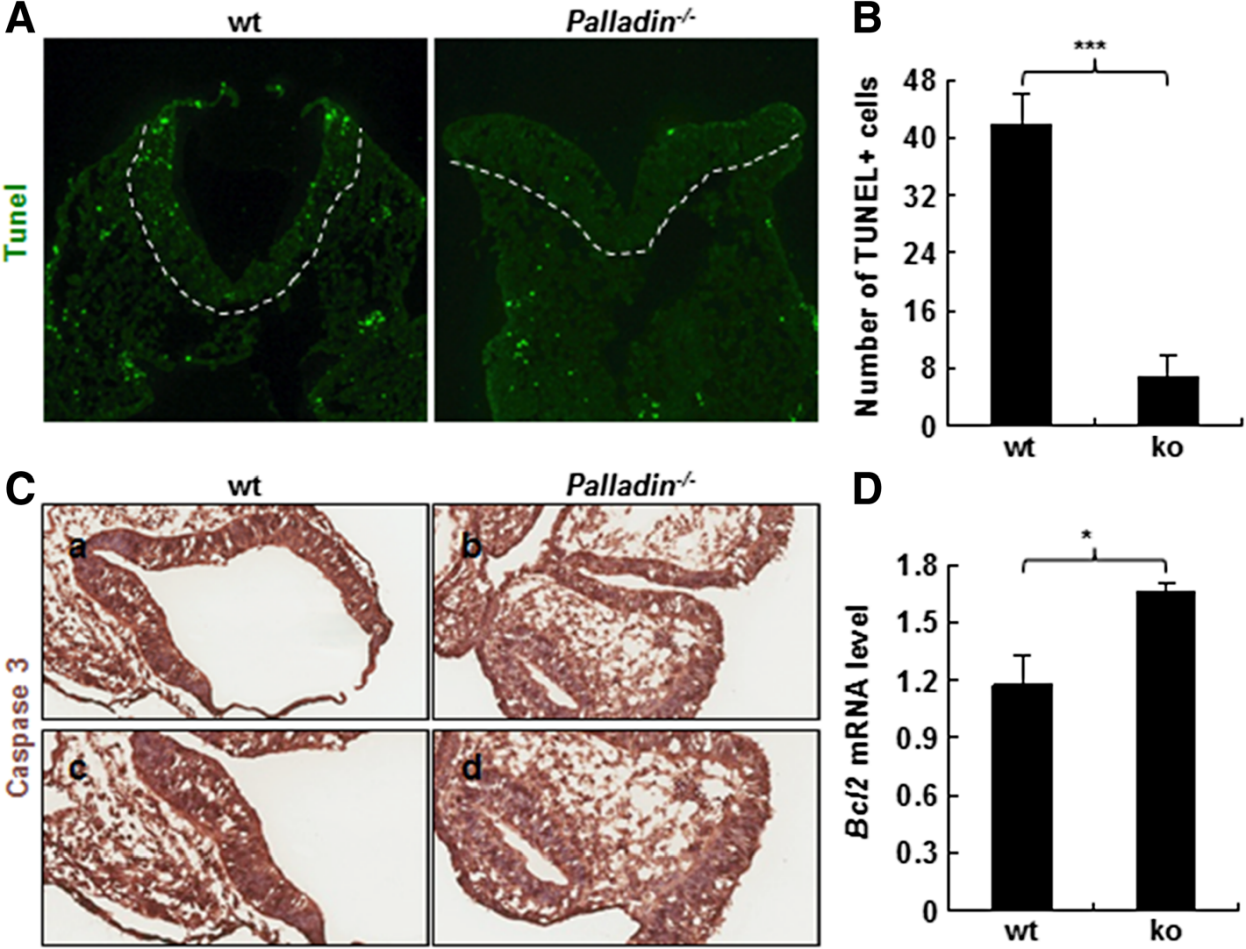
Cell apoptosis is decreased in *Palladin* ^*-/-*^ neural tube. **A** TUNEL assay shows decreased apoptosis in *Palladin* ^*-/-*^ neural tube at E9.5. **B** Quantification of TUNEL positive cells/section from the neural tube region. **C** E9.5 neural tubes are detected for Caspase 3 by IHC. Apoptosis is decreased in *Palladin* ^*-/-*^ neural tube. The neural fold region in *a* and *b* are shown at higher magnification in *c* and *d* respectively. **D** mRNA level of *Bcl2* is up-regulated in *Palladin* ^*-/-*^ embryo brain. White dotted lines outline the neural tube. Error bars indicate SEM; *P < 0.05, ***P < 0.001

Apoptosis plays an important role in NTC, tips of dorsal neural folds undergo apoptosis process so that the neural folds can bend and both ends can meet and fuse [45]. To investigate whether NTD in *Palladin* ^*-/-*^ embryos was related to a change in apoptosis in the neural fold region, TUNEL assay was performed on E9.5 cryosections. It revealed that *Palladin* ^*-/-*^ neural folds had a lower level of apoptotic cells compared with that in wt neural folds (Fig. 5A). TUNEL positive cells were quantitated, and there was a significant decrease in *Palladin* ^*-/-*^ neural tube (Fig. 5B). To confirm this finding, IHC for the antibody of caspase 3 was performed. It turned out a reduction in *Palladin* ^*-/-*^ neural tubes (Fig. 5C).

To further explore the mechanism of decreased apoptosis, we detected key regulators of cell apoptosis by qRT-PCR on E9.5 embryonic brain cDNAs. Genes have been detected included *Bcl2*, *Bcl-xl*, *Bad* and *Bax. Bcl2*, a negative regulator of apoptosis, was significantly increased in *Palladin* ^*-/-*^ embryos (Fig. 5D, Additional file 3: Figure S3D).

### Reduced cell adhesion in *Palladin*^*-/-*^ neural tubes

**Fig 6:**
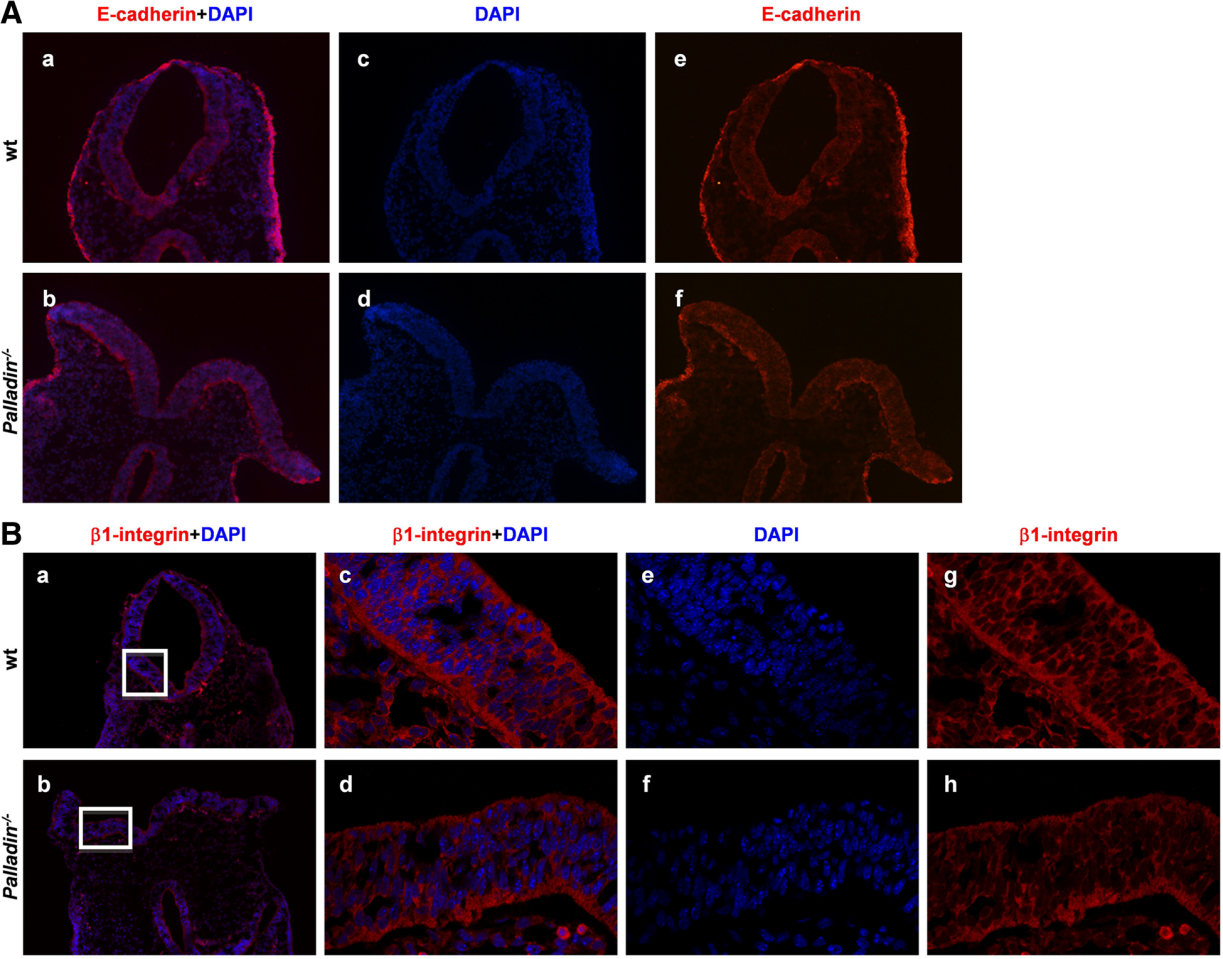
Cell adhesion shows a reduction in *Palladin* ^*-/-*^ neural tube but not in non-neural ectoderm. (**A**) Cross-sections of wt and *Palladin* ^*-/-*^ embryos at E9.5 are stained with an antibody against E-cadherin to detect cell adhesion in the surface ectoderm. There is strong expression of E-cadherin at the edge of neural folds in both wt and *Palladin* ^*-/-*^ embryos. (**B**) Wt and *Palladin* ^*-/-*^ embryos at E9.5 are stained with an antibody against β1-integrin. Cell adhesion is reduced in *Palladin* ^*-/-*^ neural tubes (*a*, *c*, *g*) than that in wt ones (*b*, *d*, *h*). The boxed regions in *a* and *b* are shown at higher magnification in *c* and *d*, respectively

Cell adhesion is another important factor that regulates NTC. During the NTC process, after the formation of dorsolateral hinge point (DLHP), neural epithelium migrate from the lateral aspects of the neural tube towards the midline, neural folds then fuse at their dorsal tips to generate a closed neural tube [45]. We have previously observed that MEFs lacking of palladin showed decreased adhesion to fibronectin compared to wt MEFs [17], so we speculated that cell adhesion in *Palladin* ^*-/-*^ neural folds would be compromised. To assess whether palladin deficiency impaired cell adhesion on the tips of the neural folds, the expression of E-cadherin (a key component of cell adhesion) was first examined by immunofluorescence staining on E9.5 cryosections. A strong expression of E-cadherin could be detected on the tips of neural folds in both wt and *Palladin* ^*-/-*^ embryos, showing no significant difference (Fig. 6A).

We previously found that β1-integrin is significantly decreased in *Palladin* ^*-/-*^ MEFs [46]. Given that integrins are critical for cell adhesion and migration [47, 48], we had reasons to presume that β1-integrin might reduce in *Palladin* ^*-/-*^ neural tube. We checked the expression of β1-integrin on embryos at E9.5 by immunofluorescence staining, and it turned out to be a decreased β1-integrin in *Palladin* ^*-/-*^ neural tube (Fig. 6B).

## Discussion

Although the function of palladin on actin organization, cancer development and invasion has been described, also molecular associations of palladin have been identified one by one, little is known about the role palladin plays during NTC process. In this paper, we show how palladin regulates NTC in vivo. We found that deletion of palladin causes changes of multiple cellular events which are critical for NTC in the developing neural folds. Actin stress fibers within the neuroepithelium is fainter. Proliferation of neural epithelium is increased. Differentiation of neural progenitors is decreased and abnormal spatially. Apoptosis is decreased. Our results suggest that palladin may regulate NTC by modulating these cellular events in the developing neural folds. Hence a general genetic pathway from palladin to the control of NTC has been established. Moreover, we find expression changes of critical molecules for each cellular events, it is absolutely useful for the establishment of direct biochemical pathway for palladin to control of NTC.

Research on expression analysis of *palladin* has been described [31], we also have investigated stage- and tissue-specific expression of *palladin* in early embryos [17], However, no reports indicate the specific cell types *palladin* is expressed during NTC, which is an important question for the study of palladin in NTC. Therefore, we perform qRT-PCR and WISH, and identify that the expression of *palladin* is highly restricted to the neural epithelium in the embryonic brain at E9.5. This indicates the particular and crucial role of palladin during NTC process. Therefore, our research focuses on the neural folds from E9.5 to E10.5.

Palladin is a cytoskeleton-associated protein. Research in vitro indicated that palladin plays a critical role in actin organization, our results confirmed this finding in vivo. Cytoskeleton is fundamental for many cellular processes, including cell proliferation and differentiation. Since loss of palladin weakens actin network in the developing neural folds, combined the phenotype of extensive outgrowth of neural epithelium in *Palladin* ^*-/-*^ embryos, it is an expected finding that proliferation is increased in palladin deficient embryos. Since proliferation is often related to cell cycle, shortened cell cycle length is probably a reason of increased proliferation in neural epithelium, thus the cell cycle length was qualified in vivo by cumulative BrdU assay. It turns out a shorter cell cycle length in palladin deficient neural progenitors, which explains the phenomenon of increased proliferation. A report has showed that down-regulation of palladin enhances cell vitality and proliferation in vitro [49], which confirms our finding. However the mechanism of palladin involving in regulating proliferation and cell cycle has not yet been studied, a better understanding of this field is necessary.

The fates of neural progenitors in the ventral side of neural folds in the early embryogenesis are proliferation and differentiation. Since proliferation is increased in *Palladin* ^*-/-*^ neural progenitors, it is possible that differentiation in the corresponding location is decreased. To confirm this hypothesis, we quantify the ratio of proliferating neural progenitors to differentiated neurons. It turns out a significant increased ratio in palladin deficient neural tube compared to that of wt. It should be noted that in the dorsal side of neural folds, differentiated neurons is increased in palladin deficient neural tube. This is probably a consequence of disturbed cytoskeleton within the neural tube, by differentiated neurons moving from the ventral side to the dorsal side. There is a report suggests that *palladin* knockdown may facilitate cell differentiation in vitro [49]. It is commonly accepted that dramatic cytoskeletal changes can trigger skeletal muscle differentiation. However, more research is needed to address whether the function of palladin in regulating differentiation is positive or negative.

In the dorsal side of neural folds, neural epithelium undergoes apoptosis to allow the two ends of neural folds to meet and fuse. However, proliferation is increased along the whole neural tube in palladin deficient embryos. So we examine apoptosis in the dorsal side of neural folds, it turns out a significant decreased apoptosis in palladin deficient neural folds. So far, little is known on the role palladin plays in cell apoptosis. For this reason, more research is needed to complete this field.

In our previous study, palladin plays an important role in regulating cell adhesion either in vivo or in vitro by stabilizing β1-integrin [17, 46]. In addition, other researches confirm this finding [50, 51]. So we examined the expression of β1-integrin in neural tube, and found it decreased slightly in palladin deficient neural tubes. Nevertheless, cell adhesion on the tips of neural folds is found to be normal in palladin deficient mouse embryos. It is possible that in surface ectoderm, the function of palladin in cell adhesion is compensated by other proteins in vivo during mouse NTC. Myotilin and myopalladin are members of the palladin/myotilin/myopalladin family [34]. Therefore, further study is needed to determine whether myotilin and myopalladin can functionally compensate for the loss of palladin in vivo during NTC.

NTC is an extremely complex process during embryogenesis. Other than proliferation, differentiation, apoptosis and adhesion, there are other events involved in NTC process. Correct head mesenchyme behaviors, correct cell division direction of non-neural ectoderm, intrinsic forces in neural plate are important for NTC [1, 52, 53]. Therefore, to better understand the mechanism of palladin regulating NTC, the function of palladin in these events requires further exploration.

## Conclusions

In conclusion, our results demonstrate that actin-associated protein palladin plays a role in cell proliferation, differentiation and apoptosis in the developing neural folds to regulate NTC process. Our work provides an evidence that palladin is a cell-cycle regulator spatially and temporally required for the neural epithelium proliferation. We believe our findings are helpful for further study of the function of palladin and a better understanding of the underlying molecular mechanism involved in NTC process.

## Additional files

Additional file 1: Figure S1. Neural patterning is normal in *Palladin* ^*-/-*^ embryos. Whole mount and section ISH of wt and *Palladin* ^*-/-*^ embryos for Twist and Otx2 at E9.5. (A) Twist is located in the head mesenchyme region in both wt (a, c, e) and *Palladin* ^*-/-*^ embryos (b, d, f). (B) Otx2 is located in the forebrain and midbrain region in both wt (a, c, e) and *Palladin* ^*-/-*^ embryos (b, d, f). The boxed regions in c and d are shown at higher magnification in e and f. (TIF 7155 kb)

Additional file 2: Figure S2. Palladin isoform 2 is the main isoform expressed in E9.5 mouse brain. Detection of Palladin isoforms in E9.5 wt mouse brain by qRT-PCR using isoform-specific primers. Palladin isoform 2 is the main expressing isoform. Isoform 3 and 4 were detected in a much lower expression level. Isoform 1 and 5 were barely detected. (TIF 284 kb)

Additional file 3: Figure S3. mRNA level changes in *Palladin* ^*-/-*^ brain. mRNA levels of *Eps8, VASP, AKT1, Profilin, LASP1, Clp36, Spin90, Ezrin, ILKAP, LPP* and *Src* showed no remarkable difference between wt and *Palladin* ^*-/-*^ brain at E9.5 (A). mRNA levels of neural progenitor genes *Sox9*, *Nestin* and *Pax6* and differentiation genes *MAP2*, *Olig2* and *Neurogenin2*, expression of *Sox9* increased in *Palladin* ^*-/-*^ embryos at E10.5 (B). Expression of *Cyclin A, Cyclin B* and *Cyclin D1* showed no significant change in *Palladin* ^*-/-*^ brain at E10.5 as assayed by qPCR (C). mRNA expression of BCL-XL, Bax and Bad in wt and *Palladin* ^*-/-*^ embryonic brains. They show no significant differences between wt and *Palladin* ^*-/-*^. Error bars indicate SEM; *P < 0.05. (TIF 1172 kb)

Additional file 4: Table S1. Antibody list. (DOCX 57 kb)

## Abbreviations

ATRA: All-trans retinoic acid
CNS: Central nervous system
DIG: Digoxigenin
DLHP: Dorsolateral hinge point
E: Embryonic day
MEF: Mouse embryonic fibroblast
NTC: Neural tube closure
NTDs: Neural tube defects
SJTUSM: Shanghai Jiao Tong University School of Medicine
WISH: Whole mount in situ hybridization
