# Supplementary figures and images for: Lacking of palladin leads to multiple cellular events changes which contribute to NTD
Source: Neural Dev. 2017 Mar 24;12:4. doi: 10.1186/s13064-017-0081-6 (PMC5366166; doi:10.1186/s13064-017-0081-6)

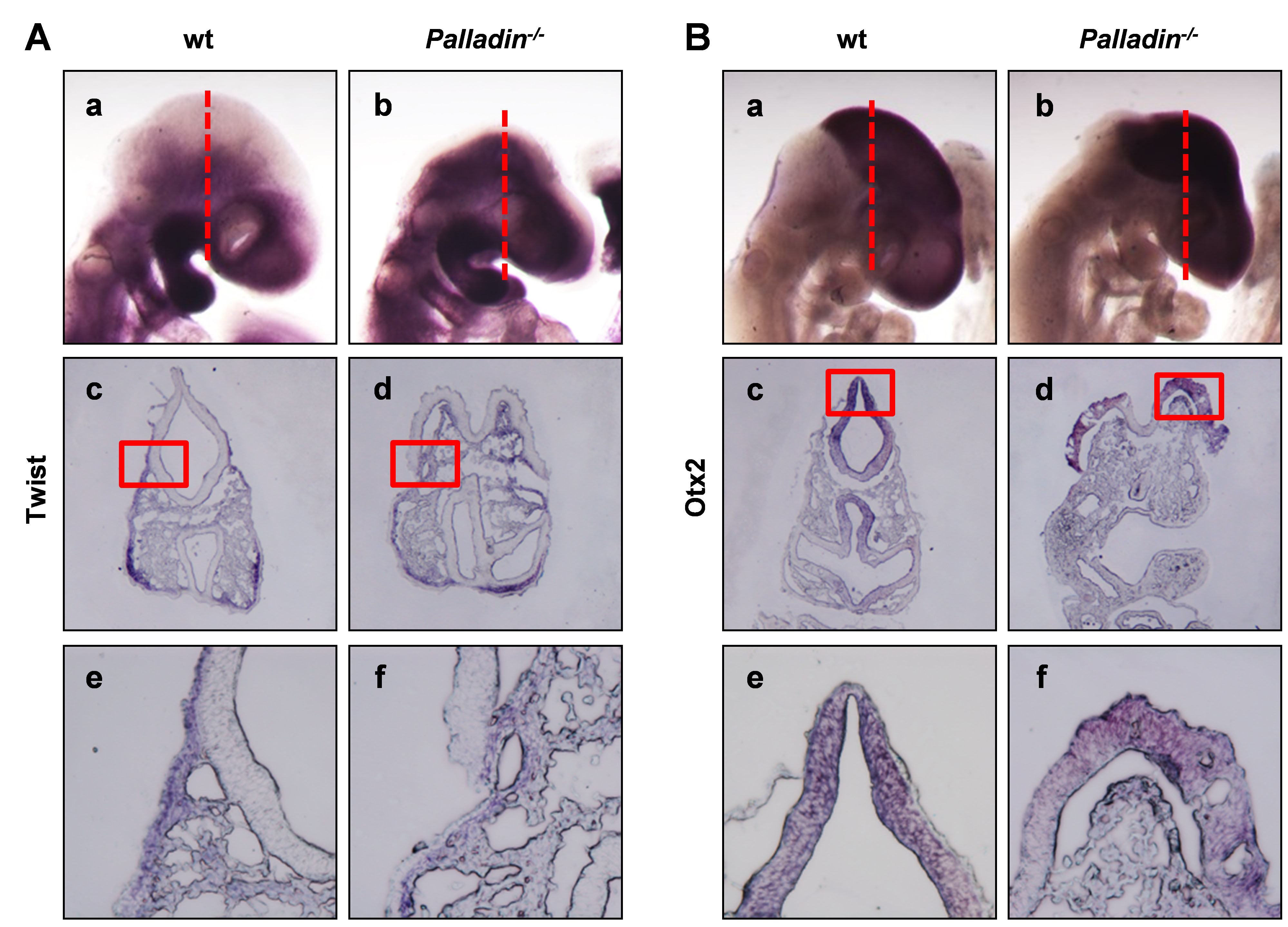

Supplement: Supplementary file 1 — Neural patterning is normal in Palladin -/- embryos. Whole mount and section ISH of wt and Palladin -/- embryos for Twist and Otx2 at E9.5. (A) Twist is located in the head mesenchyme region in both wt (a, c, e) and Palladin -/- embryos (b, d, f). (B) Otx2 is located in the forebrain and midbrain region in both wt (a, c, e) and Palladin -/- embryos (b, d, f). The boxed regions in c and d are shown at higher magnification in e and f. (TIF 7155 kb) [file 13064_2017_81_MOESM1_ESM.tif]

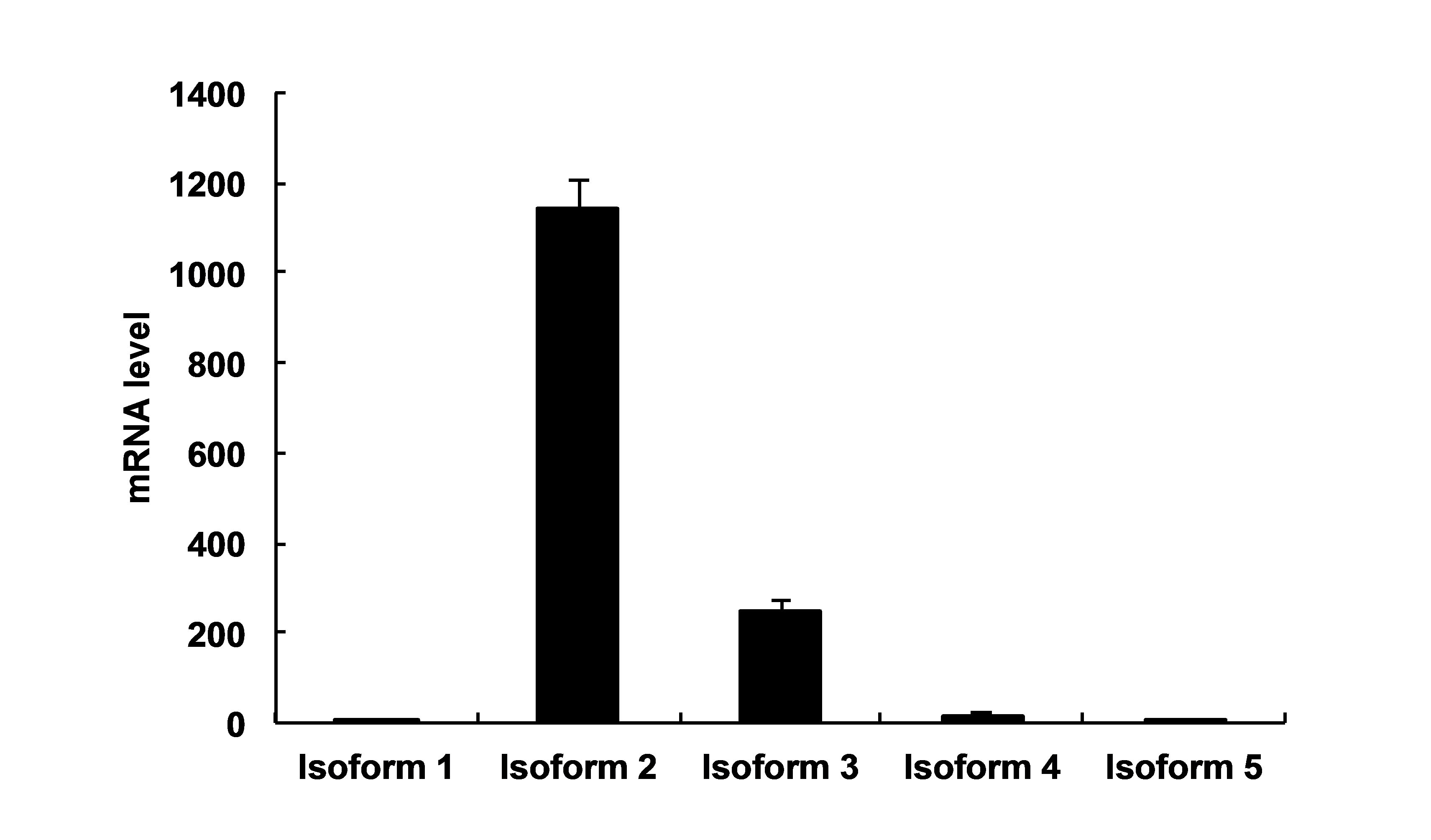

Supplement: Supplementary file 2 — Palladin isoform 2 is the main isoform expressed in E9.5 mouse brain. Detection of Palladin isoforms in E9.5 wt mouse brain by qRT-PCR using isoform-specific primers. Palladin isoform 2 is the main expressing isoform. Isoform 3 and 4 were detected in a much lower expression level. Isoform 1 and 5 were barely detected. (TIF 284 kb) [file 13064_2017_81_MOESM2_ESM.tif]

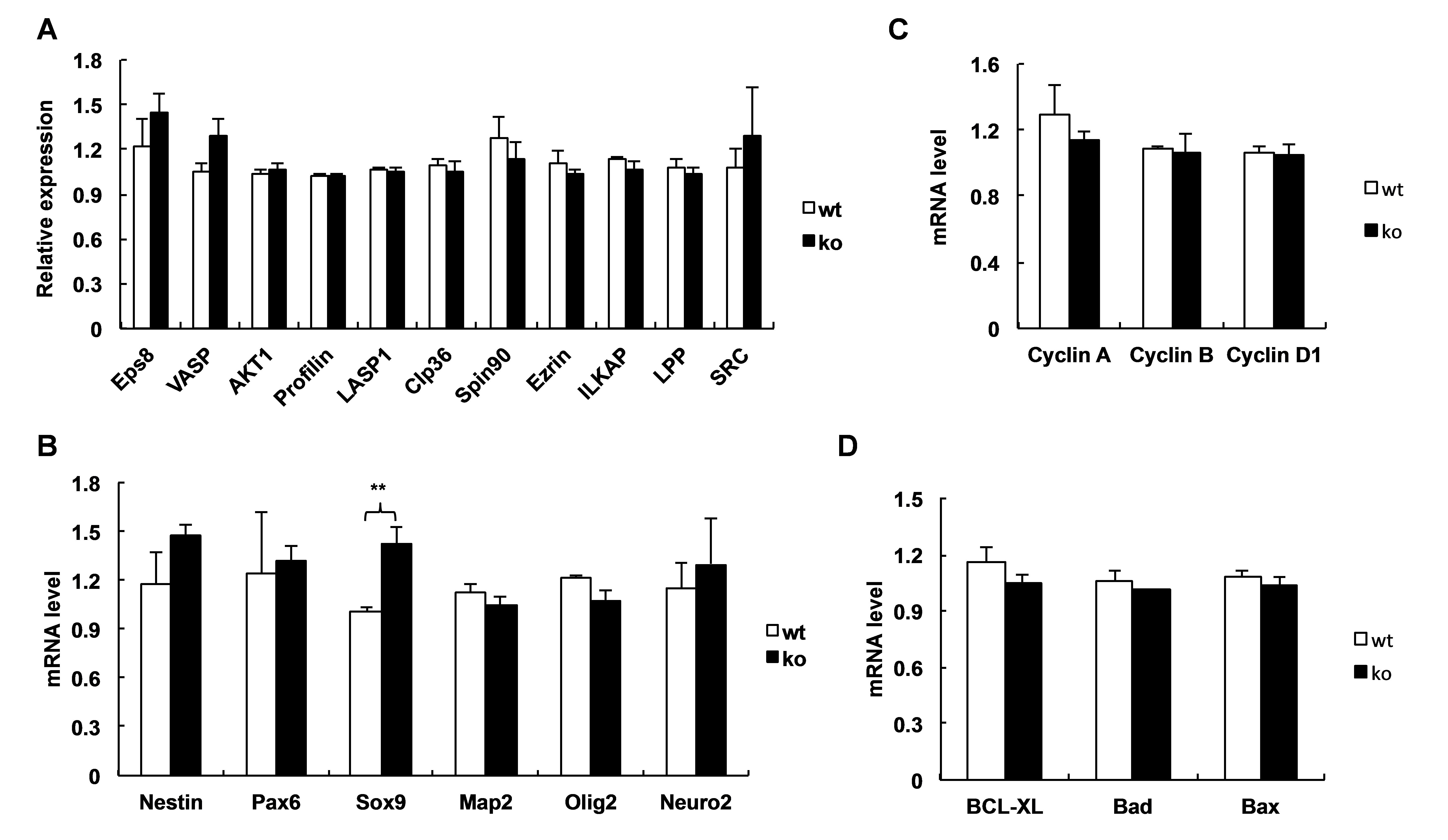

Supplement: Supplementary file 3 — mRNA level changes in Palladin -/- brain. mRNA levels of Eps8, VASP, AKT1, Profilin, LASP1, Clp36, Spin90, Ezrin, ILKAP, LPP and Src showed no remarkable difference between wt and Palladin -/- brain at E9.5 (A). mRNA levels of neural progenitor genes Sox9, Nestin and Pax6 and differentiation genes MAP2, Olig2 and Neurogenin2, expression of Sox9 increased in Palladin -/- embryos at E10.5 (B). Expression of Cyclin A, Cyclin B and Cyclin D1 showed no significant change in Palladin -/- brain at E10.5 as assayed by qPCR (C). mRNA expression of BCL-XL, Bax and Bad in wt and Palladin -/- embryonic brains. They show no significant differences between wt and Palladin -/-. Error bars indicate SEM; *P < 0.05. (TIF 1172 kb) [file 13064_2017_81_MOESM3_ESM.tif]
