# Supplementary material for: Lacking of palladin leads to multiple cellular events changes which contribute to NTD
Source: Neural Dev. 2017 Mar 24;12:4. doi: 10.1186/s13064-017-0081-6 (PMC5366166; doi:10.1186/s13064-017-0081-6)
Supplement: Supplementary file 4 — Antibody list. (DOCX 57 kb) [file 13064_2017_81_MOESM4_ESM.docx]

**Table S1. Antibody list**

| **Antibody** | **Supplier** | **Catalog number** | **Dilution for IF** |
| --- | --- | --- | --- |
| PH3 | Millipore | 06-570 | 1:300 |
| Ki67 | Thermo Scientific | RB-1510-P | 1:100 |
| Tuj1 | R&D | MAB1195 | 1:100 |
| Sox9 | Millipore | AB5535 | 1:300 |
| P27 | Cell signal technology | 3698 | 1:1000 |
| BrdU | Thermo Scientific | MS-1058-B | 1:100 |
| Phalloidin | Invitrogen | A12379 | 1:800 |
| E-cadherin | BD | 610181 | 1:100 |
| Nestin | Santa cruz | Sc-20978 | 1:100 |
| Palladin | Proteintech | 10853-1-AP | 1:150 |
| a-actinin | Sigma-aldrich | A5044 | 1:200 |
| β1-integrin | Cell signal technology | 9699 | 1:500 |
